# Effect of sex and polymorphisms of CYP2B6 and UGT1A9 on the difference between the target-controlled infusion predicted and measured plasma propofol concentration

**DOI:** 10.1186/s40981-018-0196-8

**Published:** 2018-08-13

**Authors:** Ai Fujita, Kengo Hayamizu, Tatsuya Yoshihara, Masayoshi Zaitsu, Fumie Shiraishi, Hisatomi Arima, Kazumasa Matsuo, Kanako Shiokawa, Hidekazu Setoguchi, Toshiyuki Sasaguri

**Affiliations:** 1 Department of Clinical Pharmacology, Faculty of Medical Sciences, Kyushu University, Maidashi 3-1-1, Higashi-ku, Fukuoka, 812-8582 Japan; 2 Department of Anesthesiology, Chihaya Hospital, Chihaya 2-30-1, Higashi-ku, Fukuoka, Japan; 3 Department of Anesthesiology and Critical Care Medicine, Faculty of Medical Sciences, Kyushu University, Maidashi 3-1-1, Higashi-ku, Fukuoka, 812-8582 Japan; 4 Clinical Research Center, Fukuoka Mirai Hospital, Kashiiteriha 3-5-1, Higashi-ku, Fukuoka, 813-0017 Japan; 5 Department of Social and Behavioral Sciences, Harvard T.H. Chan School of Public Health, 677 Huntington Ave, Boston, MA 02115 USA; 6 Department of Public Health, Graduate School of Medicine, The University of Tokyo, Hongo 7-3-1, Bunkyo-ku, Tokyo, 113-0033 Japan; 7 Department of Preventive Medicine and Public Health, Faculty of Medicine, Fukuoka University, Nanakuma 8-19-1, Jonan-ku, Fukuoka, 814-0180 Japan; 8 Department of Anesthesiology, Kyushu Medical Center, Jigyohama 1-8-1, Chuo-ku, Fukuoka, 810-8563 Japan; 9 Yamamoto Memorial Hospital, Hachiyagarami 88-4, Niricho, Imari, Saga, 848-0031 Japan

**Keywords:** Propofol, Target-controlled infusion, Polymorphism, Sex difference, Mean blood pressure, Heart rate, Volume of intravenous fluid

## Abstract

**Introduction:**

To examine whether sex and polymorphisms of cytochrome P450 (CYP) 2B6 and UDP-glucuronosyltransferase (UGT) 1A9 affect the difference between predicted and measured plasma propofol concentration during continuous infusion by target-controlled infusion.

**Results:**

Blood samples of 69 patients (48 men and 21 women) were obtained at 4 h after initial propofol infusion. Percentage performance error (PE) was calculated to assess the difference between measured and predicted propofol concentration. Regression coefficients (β) and 95% confidence intervals (CI) of sex and the polymorphisms of CYP2B6 and UGT1A9 for PE were, separately and mutually, estimated with linear regression. Covariates included age and body mass index in the minimal adjusted model, and additionally included clinical factors (mean blood pressure, heart rate, volume of intravenous fluid, surgical site, surgical position, and pneumoperitoneum) in the full adjusted model. PE was higher in men than in women (28.7% versus 10.5%, *p =* 0.015). Female sex was inversely associated with PE: the minimal adjusted β = − 8.84 (95% CI, − 16.26 to − 1.43); however, the fully adjusted β with clinical factors became not significant. The average of PE did not differ between polymorphisms of CYP2B6 and UGT1A9, and β of CYP2B6 516G>T polymorphisms mutually adjusted with female sex was not significant. Mean blood pressure, heart rate, and volume of intravenous fluid were independently associated with PE in the full adjusted model.

**Conclusions:**

Under 4 h anesthesia with propofol target-controlled infusion in our population, sex differences appeared to exist in the propofol concentration, which might be largely mediated by clinical factors, such as hemodynamic status.

**Trial registration:**

UMIN-CTR UMIN000009015, Registered 1 October 2012

**Electronic supplementary material:**

The online version of this article (10.1186/s40981-018-0196-8) contains supplementary material, which is available to authorized users.

## Background

Propofol (2,6-diisopropyl-phenol) is commonly used in induction and maintenance of general anesthesia due to its rapid onset, relatively short emergence time, and favorable safety profile. The target-controlled infusion (TCI) system is widely used to administer propofol. Diprifusor™ is a TCI system that automatically regulates propofol dose using a pharmacokinetic model to achieve a target blood concentration [1]. Body weight is used as an input parameter for Diprifusor™. Predicted plasma propofol concentration (Cp) and measured plasma propofol concentration (Cm) were found to be correlated, in spite of the slightly greater values of Cm compared to Cp [1–4]. If Cm is higher than Cp, arousal delay may occur; if Cm is lower than Cp, it may lead to intraoperative body movement and awakening during surgery, which may result in poor prognosis for the patient.

Regarding the pharmacokinetics of propofol, sex has been reported to influence propofol metabolism and the effect of propofol, in addition to age, height, and weight [5–15]: for example, propofol metabolism in women is faster than that in men, and women awake faster than men. For clinical factors, hemodynamic state might also affect the accuracy of the TCI system [16], because the clearance of propofol depends on the hepatic blood flow [17–19].

In addition, genetic polymorphisms of the propofol metabolism enzymes have been shown to affect propofol blood concentration. The biotransformation of propofol is greatly dependent on liver metabolism [17–19], and cytochrome P450 (CYP) 2B6 and uridine diphosphate (UDP)-glucuronosyltransferase (UGT) 1A9, the main enzymes involved in propofol metabolism, are responsible for the hydroxylation and glucuronidation of propofol [20–22]. Single-nucleotide polymorphisms (SNPs) in CYP2B6 and UGT1A9 might contribute to the inter-individual variability in the rate of formation of propofol metabolites [21–23], while several studies reported no significant effect of these SNPs on propofol metabolism [7, 8, 10, 24].

Although previous studies investigated the contribution of sex and polymorphisms of propofol metabolizing enzymes on propofol blood concentration under single intravenous bolus or short-term propofol infusion [7, 8, 10–16], the influence of those factors on the accuracy of Diprifusor™ during long-term propofol infusion remains unclear.

Herein, we examined whether sex and polymorphisms of CYP2B6 and UGT1A9 affect the difference between the predicted concentration and actual plasma propofol concentration in the perioperative period with continuous propofol infusion of 4 h.

## Methods

### Main outcome and patient selection

The main outcome of the study was the difference between Cp and Cm around the maintenance concentration of propofol in surgery, which was evaluated using percentage performance error (PE) as follows: PE (%) = (Cm - Cp)/Cp × 100.

We included patients aged ≥ 20 years, underwent anesthesia (expected anesthesia time ≥ 4 h) in spine and lateral positions by administration of propofol by TCI, and monitored with direct arterial blood pressure. We excluded those with anemia (hemoglobin < 10 g/dl), liver dysfunction (Child-Pugh B or C), renal dysfunction (eGFR < 30 ml/min/1.73m^2^), American Society of Anesthesiologists-physical status (ASA-PS) class III/IV, hepatic or renal surgery, and psychoneurotic disorders or psychiatric pharmacotherapy.

Of 70 eligible patients, we excluded one outlier of 39-year-old woman with a great discrepancy in Cp and Cm (PE = 267.9%) due to a possible technical error; 69 patients (48 men and 21 women) comprised the study subject. A prior analysis included the excluded patient showed the similar trend (data not shown).

### Measurement of propofol concentration and clinical parameters

Anesthesia was induced and maintained with continuous infusion of remifentanil and propofol. A propofol TCI system (TE-371, TERUMO, Tokyo, Japan) was used to administer propofol. The infusion rates of propofol and remifentanil were adjusted by the anesthesiologists in charge according to the patients’ condition. Direct arterial blood pressure, heart rate (HR), ECG, SpO_2_, central core temperature, and end tidal CO_2_ were recorded throughout all operations. Bispectral index (BIS, QE-910P, Nihon Kohden, Tokyo, Japan) were applied unless it did not disturb the procedure of the surgeries.

Blood samples were collected from the radial artery at 4 h after initial propofol infusion after matching predicted blood concentration and effect-site concentration displayed on the TCI devices. When the duration of propofol infusion was < 4 h, the sample was collected before changing the target blood concentration. Mean blood pressure (mBP) and HR were recorded at the time of sample collection. Total volume of intravenous fluid was measured from initial propofol infusion to blood sample collection. Blood samples were used for gene polymorphism analysis and measurement of plasma propofol concentration. Although the time of blood pressure before blood sample collection might affect the concentration of propofol, a prior analysis using blood pressure of 10 min before blood sampling showed the same results (data not shown).

The plasma concentration of propofol was determined by a modified method of a previous report [25] by a commercial laboratory, BML, Inc. (Tokyo, Japan), using a reverse phase high-performance liquid chromatography system (Hitachi High-Technologies Corporation, Tokyo, Japan, and Shimadzu Corporation, Kyoto, Japan) with a Hypersil C18 reversed-phase column (3 μm particle size, 100 × 5.0 mm I.D.). The excitation and emission wavelength were 276 and 310 nm, respectively. Blood samples were centrifuged (1150 *g* for 10 min) and stored at 4 °C. A calibration graph was created by plotting the ratios of the areas for propofol to those for thymol (internal standard) from 0.2 to 5 μg/ml. The limit of quantitation was 0.1 μg/ml.

### Genotyping

Genomic DNA was extracted from peripheral blood with a DNA isolation kit (GenTLE; Takara Bio, Ohtsu, Japan). Genotyping of CYP2B6 499 C > G (rs3826711), 785 A > G (rs2279343), 1375 A > G, and 1459 C > T (rs3211371) was performed by polymerase chain reaction with restriction fragment length polymorphism method. CYP2B6 516 G > T (rs3745274), UGT1A9 i399C > T (rs2741049), and 766 G > A (rs58597806) SNPs were identified by validated TaqMan SNP Genotyping Assays (assay ID: C_7817765_60, C_34816143_20, and C_9096281_10, respectively) (Life Technologies, Carlsbad, California, USA) using an ABI 7500 Real-Time PCR system (Life Technologies) and TaqMan®Universal Master Mix II with UNG (Life Technologies) according to the manufacturer’s instruction. CYP2B6 and UGT1A9 genotypes were determined by an investigator blinded to individual information. The observed allelic frequencies conformed to Hardy-Weinberg equilibrium (data not shown).

### Statistical analysis

To assess the contribution of sex and polymorphisms for PE separately, crude regression coefficients (β) and 95% confidence intervals (CI) of female sex and CYP2B6 516G > T polymorphisms were, respectively, estimated with linear regression (model 1). In addition, we minimally adjusted for baseline characteristics (age and BMI) in model 2 and fully adjusted for baseline characteristics (age and BMI) and clinical factors (mBP, HR, volume of intravenous fluid, surgical site, head down position, pneumoperitoneum) in model 3. Those covariates were adjusted as mediators for the association; other clinical factors, such as BIS scores and body temperature, were not included for covariates because those did not affect sex differences of PE in a prior analysis (Additional file 1: Table S1).

In mutual adjusted models, we included explanatory variables of female sex and CYP2B6 516G > T polymorphisms simultaneously (model 4). Additionally, we minimally adjusted for baseline characteristics (model 5) and fully adjusted for baseline characteristics and clinical factors (model 6). In the mutual adjusted models, due to the lack of female patients with CYP2B6 516TT polymorphisms, we excluded three male patients with CYP2B6 516TT polymorphisms.

Alpha was set at 0.05, and all *p* values were two-sided. Data were analyzed using JMP Pro 11.0.0 (SAS Institute Inc., Tokyo, Japan).

## Results

**Fig. 1:**
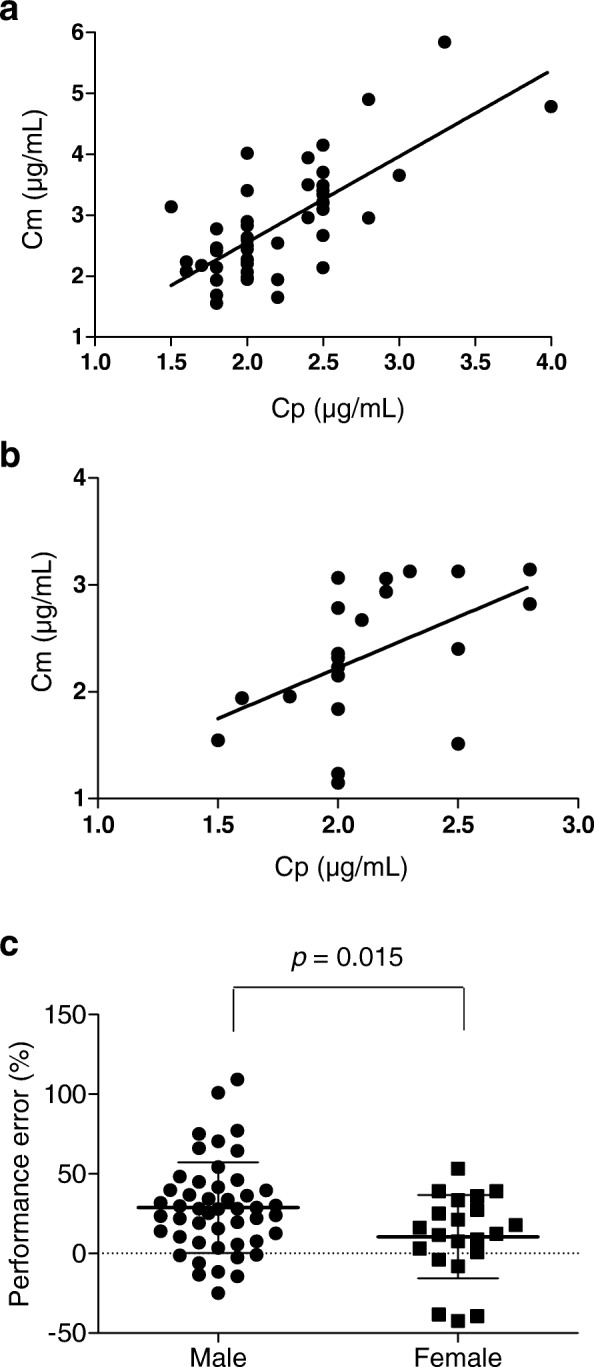
**a**, **b** Correlation of predicted plasma propofol concentration (Cp) and measured plasma propofol concentration (Cm) by sex: **a** men and **b** women. **c** Differences of performance error by sex

The baseline characteristics and clinical factors did not differ between men and women (Table 1). Cp and Cm were statistically correlated: the correlation coefficients were, respectively, 0.73 in men (*p* < 0.001, Fig. 1a) and 0.50 in women (*p* < 0.001, Fig. 1b). The average of PE in men was significantly higher than that in women (28.7% versus 10.5%, *p* = 0.015, Table 1 and Fig. 1c).

**Table 1:** Baseline characteristics and clinical factors of the study subjects stratified by sex

Characteristics	Male (*n* = 48) Mean ± SD or number (%)	Female (*n* = 21) Mean ± SD or number (%)	*p* value^a^
Performance error (%)	28.7 ± 28.5	10.5 ± 26.2	0.015
Age (years)	66.0 ± 10.7	66.5 ± 11.1	0.872
BMI (kg/m^2^)	23.3 ± 2.9	22.5 ± 2.4	0.279
CYP2B6 516G > T			
GG	35 (73)	13 (62)	0.198
GT	10 (21)	10 (38)	
TT	31 (6)	0 (0)	
Volume of intravenous fluid (mL/kg)	31.5 ± 11.9	32.4 ± 13.7	0.773
mBP (mmHg)	67.1 ± 8.9	63.8 ± 8.5	0.160
HR (bpm)	62.8 ± 11.2	67.8 ± 11.6	0.096
Surgical site (*n*)			
Head and neck/upper abdomen/lower abdomen	25/ 9/ 15	16/ 5/ 0	0.015
Surgical position (*n*)			
Flat position/head down position	37/ 11	21/ 0	0.017
Pneumoperitoneum (*n*)	3	2	0.629

^a^*t* test or chi-square test

Volume of intravenous fluid means the total infusion volume from commencement of propofol infusion to the time of blood sample collection. Blood pressure and heart rate were recorded at the time of blood sample collection. *BMI* body mass index, *mBP* mean blood pressure, *HR* heart rate

The results of CYP2B6 and UGT1A9 SNPs analyses are shown in Table 2 and Additional file 2: Table S2. Although the allele frequencies for any polymorphisms did not statistically differ between men and women (Additional file 2: Table S2), the average of PE tended to differ between CYP2B6 516G > T polymorphisms (*p* = 0.050, Table 2).

**Table 2:** Mean values and standard deviation of performance error stratified by polymorphisms

Polymorphisms	Performance error (%) Means ± SD					*p* value^a^
CYP2B6						
499C > G	CC (*n* = 68)	CG (*n* = 1)	–			–
	23.4 ± 29.1	10.4				
516G > T	GG (*n* = 48)	GT (*n* = 18)	TT (*n* = 3)			
	26.6 ± 29.8	10.4 ± 23.5	45.0 ± 21.2			0.050
785A > G	AA (*n* = 36)	AG (*n* = 27)	GG (*n* = 6)			
	23.9 ± 29.3	19.7 ± 30.3	34.2 ± 19.9			0.534
1375A > G	AA (*n* = 68)	AG (*n* = 1)				
	22.0 ± 27.5	100.9		–		–
1459C > T	CC (*n* = 65)	CT (*n* = 4)				
	22.8 ± 29.1	29.0 ± 29.9		–		0.681
UGT1A9						
i399C > T	CC (*n* = 10)	CT (*n* = 41)	TT (*n* = 18)			
	25.5 ± 22.9	21.1 ± 24.0	26.6 ± 41.1			0.776
766G > A	GG (*n* = 69)					
	23.2 ± 28.9	–	–			–

^a^Analysis of variance

In regression analysis, female sex was inversely associated with PE (Table 3): the minimal adjusted β = − 8.84 (95% CI, − 16.26 to − 1.43). However, the association was substantially attenuated and became not significant on the full-adjustment with clinical factors (model 3, Table 3). Likewise, compared with CYP2B6 516GG, CYP2B6 516G > T polymorphisms was inversely associated with PE in the crude and minimally adjusted models (Table 3). However, the association was substantially attenuated and became not significant on the full-adjustment with clinical factors (model 3, Table 3). CYP2B6 516TT polymorphisms were not associated with PE.

**Table 3:** Regression coefficients of sex and CYP2B6 516G > T polymorphisms for performance errors estimated with linear regression, adjusted for background characteristics and clinical factors

Characteristics	Beta (95% confidence interval)					
	Model 1	*p* value	Model 2^a^	*p* value	Model 3^b^	*p* value
Sex (female)	− 9.13 (− 16.40 to − 1.85)	0.015	− 8.84 (− 16.26 to − 1.43)	0.020	− 5.43 (− 12.53 to 1.68)	0.132
Age (years)			− 0.20 (− 0.88 to 0.48)	0.566	0.13 (− 0.48 to 0.73)	0.679
BMI (kg/m^2^)			0.60 (− 2.07 to 3.27)	0.656	0.53 (− 1.74 to 2.81)	0.641
mBP (mmHg)					0.80 (0.07 to 1.54)	0.033
HR (bpm)					− 0.89 (− 1.43 to − 0.34)	0.002
Volume of intravenous fluid (ml/kg)					− 0.63 (− 1.17 to − 0.09)	0.024
Surgical site						
Head and neck					Reference	
Upper abdomen					1.08 (−11.61 to 13.77)	0.865
Lower abdomen					− 4.79 (−22.73 to 13.15)	0.595
Head down position					2.98 (− 11.07 to 17.04)	0.673
Pneumoperitoneum					− 7.68 (− 20.33 to 4.96)	0.229
*R* ^2^	0.09		0.10		0.45	
CYP2B6 polymorphisms						
516GG	Reference		Reference		Reference	
516GT	− 16.93 (− 31.11 to − 2.74)	0.020	− 15.68 (− 30.55 to − 1.11)	0.036	− 8.72 (− 21.54 to 4.11)	0.179
516TT	17.65 (− 4.53 to 39.83)	0.112	15.68 (− 7.42 to 38.77)	0.180	12.12 (− 7.66 to 31.90)	0.225
Age (years)			− 0.05 (− 0.74 to 0.64)	0.884	0.20 (− 0.42 to 0.81)	0.529
BMI (kg/m^2^)			0.84 (− 1.89 to 3.57)	0.542	0.62 (− 1.70 to 2.94)	0.594
mBP (mmHg)					0.98 (0.26 to 1.71)	0.009
HR (bpm)					− 0.90 (− 1.45 to − 0.34)	0.002
Volume of intravenous fluid (ml/kg)					− 0.62 (− 1.17 to − 0.08)	0.027
Surgical site						
Head and neck					Reference	
Upper abdomen					0.56 (− 12.31 to 13.43)	0.931
Lower abdomen					− 0.67 (− 18.06 to 16.72)	0.939
Head down position					2.37 (− 11.85 to 16.58)	0.740
Pneumoperitoneum					− 9.12 (− 21.69 to 3.46)	0.152
*R* ^2^	0.09		0.09		0.44	

^a^Adjusted for age and BMI (model 2)

^b^Additional adjustment for mBP, HR, volume of intravenous infusion, surgical site, head down position, and pneumoperitoneum (Model 3)

*BMI* body mass index, *mBP* mean blood pressure, *HR* heart rate

In the mutual model, β of female sex was significant, but β of CYP2B6 516G > T polymorphisms was not significant (model 4, Table 4). The association of female sex was marginally significant on the minimal adjustment with baseline characteristics (model 5, Table 4); however, the association was substantially attenuated and became not significant in the full-adjusted model with clinical factors (model 6, Table 4). The clinical factors, mBP, HR, and volume of intravenous fluid were independently associated with PE (model 3 in Table 3 and model 6 in Table 4).

**Table 4:** Regression coefficients estimated with linear regression, mutually adjusted for sex and CYP2B6 516G > T polymorphisms

Characteristics^a^	Beta (95% confidence interval)					
	Model 4^b^	*p* value	Model 5^c^	*p* value	Model 6^c^	*p* value
Sex (female)	− 7.50 (− 14.89 to − 0.12)	0.047	− 7.26 (− 14.81 to 0.29)	0.059	− 4.50 (− 12.02 to 3.02)	0.236
CYP2B6 516GT	− 6.80 (− 14.52 to 0.93)	0.084	− 6.86 (− 14.81 to 1.08)	0.089	− 2.08 (− 9.20 to 5.04)	0.560
Age (years)			0.02 (− 0.70 to 0.74)	0.946	0.17 (− 0.49 to 0.82)	0.614
BMI (kg/m^2^)			0.78 (− 1.97 to 3.53)	0.574	0.44 (− 1.96 to 2.85)	0.713
mBP (mmHg)					0.85 (0.08 to 1.63)	0.032
HR (bpm)					− 0.88 (− 1.46 to − 0.30)	0.004
Volume of intravenous fluid (ml/kg)					− 0.58 (− 1.14 to − 0.02)	0.043
Surgical site						
Head and neck					Reference	
Upper abdomen					1.36 (−11.77 to 14.49)	0.836
Lower abdomen					− 4.06 (− 22.58 to 14.46)	0.662
Head down position					2.82 (− 11.62 to 17.26)	0.697
Pneumoperitoneum					− 7.60 (− 20.59 to 5.40)	0.246
*R* ^2^	0.12		0.13		0.44	

^a^Due to the lack of women with CYP2B6 516TT in the data set, analyzed subjects were restricted to those with CYP2B6 516GG (*n* = 48) and CYP2B6 516GT (*n* = 18)

^b^Linear regression, mutually adjusted for sex and CYP2B6 516G > T polymorphisms (model 4)

^c^Additional adjustment for age and BMI (model 5); age, BMI, mBP, HR, volume of intravenous infusion, surgical site, head down position, and pneumoperitoneum (model 6)

*BMI* body mass index, *mBP* mean blood pressure, *HR* heart rate

## Discussion

This is the first study to investigate PE associated with sex and polymorphisms of CYP2B6 and UGT1A9, with clinical factors, after long-term propofol infusion. We found that PE in women was closer to 0 than that in men, which might indicate that the propofol TCI system is more accurate in women than in men.

A number of reports have suggested that the plasma concentration of propofol decreases more rapidly in women than in men, and that women tend to recover faster from propofol anesthesia than men [7–9, 11–15]. Loryan et al. suggested this sex difference was due to the fact that women’s livers have 1.9-fold greater CYP2B6 protein levels than men [7]. In this study, the mean PE in the perioperative period was significantly lower in women than in men, which might be caused by the sex difference of propofol pharmacokinetics.

The genetic background of propofol-metabolizing enzymes, CYP2B6 and UGT1A9, may vary propofol metabolite levels [20–22]; however, the association is controversial. Several studies have reported the effects of SNPs in CYP2B6 and UGT1A9 on propofol metabolism [7, 20, 21, 23, 24, 26], for example, Takahashi et al. reported that D256N polymorphism in UGT1A9 lowers enzyme activity in an in vitro study, suggesting carriers of D256N might be at risk of suffering adverse effects of propofol [21]; Kansaku et al. found that SNPs CYP2B6 G516T and UGT1A9 I399C > T determined the pharmacokinetics and pharmacodynamics of propofol [23]; and Mastrogianni et al. reported a strong trend towards the association of the CYP2B6 G516T variant with high blood propofol concentrations after a single bolus dose [26]. In contrast, other studies reported no significant effects of these SNPs on propofol metabolism [7, 8, 10, 24], which partially concur with our study.

In our study, it should be noted that clinical factors of hemodynamic status (HR and mBP), which could influence the hepatic blood flow, may substantially mediate the sex difference of PE. A higher tendency of HR in women (Table 1, *p* = 0.096) might partly explain the mediation in our study, given the fact that propofol is mostly metabolized in the liver, and the hepatic clearance of propofol depends on the hepatic blood flow [17–19]. Volume of intravenous fluid was inversely associated with PE, which could be explained by the decreased concentration of propofol due to increased plasma volume and the increased propofol metabolism rate due to increased hepatic blood flow.

This study had some limitations. First, we collected only one sample for each subject. More samples are usually collected for the external evaluation of TCI system. Second, more hemodynamic information, such as cardiac output, which can enhance liver blood flow is required. We only have the data of blood pressure and HR at the time of sample collection. Third, the target concentration of propofol was determined by the anesthesiologist in charge according to the patients’ condition because this was an observational study. Fourth, blood laboratory test was not conducted at the time of sample collection; therefore, serum albumin or hemoglobin values were not obtained. Lastly, due to the small sample size with insufficient statistical power, we could not fully assess CYP2B6 and UGT1A9 polymorphisms, in relation to sex and clinical factors. Further studies with a large number of patients are needed to clarify the influence of CYP2B6 and UGT1A9 polymorphisms on the accuracy of propofol TCI system.

## Conclusions

The accuracy of estimated propofol concentration by Diprifusor™ TCI system appeared to differ between men and women in our population. The PE in men was higher than in women which might be mainly caused by hemodynamic status. Although this study did not show significant influence of CYP2B6 and UGT1A9 polymorphisms, further studies are expected to elucidate the effect of those polymorphisms.

## Additional files

Additional file 1: **Table S1.** Other background characteristics of the study subjects stratified by sex. (DOCX 15 kb)

Additional file 2: **Table S2.** Genetic polymorphisms of CYP2B6 and UGT1A9 by stratified sex. (DOCX 14 kb)

## Abbreviations

ALT: Alanine amino transferase
ASA-PS: American Society of Anesthesiologists-physical status
BIS: Bispectral index
BMI: Body mass index
CI: Confidence interval
Cm: Measured plasma propofol concentration
Cp: Predicted plasma propofol concentration
CYP2B6: Cytochrome P450 2B6
eGFR: Estimated glomerular filtration rate
HR: Heart rate
mBP: Mean blood pressure
PE: Performance error
SNPs: Single-nucleotide polymorphisms
TCI: Target-controlled infusion
UGT1A9: Uridine diphosphate (UDP)-glucuronosyltransferase 1A9
