# Supplementary material for: Effect of sex and polymorphisms of CYP2B6 and UGT1A9 on the difference between the target-controlled infusion predicted and measured plasma propofol concentration
Source: JA Clin Rep. 2018 Aug 13;4:59. doi: 10.1186/s40981-018-0196-8 (PMC6966915; doi:10.1186/s40981-018-0196-8)
Supplement: Supplementary file 1 — Table S1. Other background characteristics of the study subjects stratified by sex. (DOCX 15 kb) [file 40981_2018_196_MOESM1_ESM.docx]

Additional file 1: Table S1. Other background characteristics of the study subjects stratified by sex

|  | Male (n=48) | | | Female (n=21) | | | P value^a^ |
| --- | --- | --- | --- | --- | --- | --- | --- |
| ASA-PS I/II (n) | 6/42 | | | 6/15 | | | 0.105 |
| Surgical position (n)  Spine/ Lateral | 39/ 9 | | | 16/ 5 | | | 0.631 |
| ALT (IU/L) | 31 | ± | 25 | 23 | ± | 20 | 0.200 |
| eGFR (mL/min/1.73 m^2^) | 72.6 | ± | 15.6 | 72.2 | ± | 21.9 | 0.928 |
| Remifentanil dosage (µg/kg/min) | 0.35 | ± | 0.16 | 0.33 | ± | 0.12 | 0.718 |
| Duration of propofol infusion (min) | 234.8 | ± | 15.9 | 232.1 | ± | 18.4 | 0.549 |
| Volume of blood loss and urine output (mL/kg) | 13.1 | ± | 13.5 | 12.0 | ± | 11.1 | 0.748 |
| BIS score  (Male: n=44; Female: n=18) | 45 | ± | 6 | 44 | ± | 7 | 0.532 |
| Body temperature (°C) | 36.9 | ± | 0.5 | 36.9 | ± | 0.6 | 0.608 |

Data are expressed as number or means ± S.D.

^a^ t-test or chi square test.

ALT and eGFR were measured before surgeries, and other clinical data were recorded at the time of blood sample collection. *ASA-PS* American Society of Anesthesiologists-physical status, *ALT* alanine amino transferase, *eGFR* estimated glomerular filtration rate, *BIS* bispectral index.
