# Supplementary material for: Effect of sex and polymorphisms of CYP2B6 and UGT1A9 on the difference between the target-controlled infusion predicted and measured plasma propofol concentration
Source: JA Clin Rep. 2018 Aug 13;4:59. doi: 10.1186/s40981-018-0196-8 (PMC6966915; doi:10.1186/s40981-018-0196-8)
Supplement: Supplementary file 2 — Table S2. Genetic polymorphisms of CYP2B6 and UGT1A9 by stratified sex. (DOCX 14 kb) [file 40981_2018_196_MOESM2_ESM.docx]

Additional file 2: Table S2. Genetic polymorphisms of CYP2B6 and UGT1A9 by stratified sex

| Polymorphisms | Male (n=48) | Female (n=21) | P value^a^ |
| --- | --- | --- | --- |
| CYP2B6  499C>G CC/CG/GG (n)  516G>T GG/GT/TT (n)  785A>G AA/AG/GG (n)  1375A>G AA/AG/GG (n)  1459C>T CC/CT/TT (n) | 47/1/0  35/10/3  26/18/4  47/1/0  46/2/0 | 21/0/0  13/8/0  10/9/2  21/0/0  19/2/0 | 0.505  0.198  0.882  0.505  0.381 |
| UGT1A9  i399C>T CC/CT/TT (n)  766G>A GG/GA/AA (n) | 8/26/14  48/0/0 | 2/15/4  21/0/0 | 0.403  - |

^a^ chi square test.
